# Early mobilization on continuous renal replacement therapy is safe and may improve filter life

**DOI:** 10.1186/cc14001

**Published:** 2014-07-28

**Authors:** Yi Tian Wang, Terry P Haines, Paul Ritchie, Craig Walker, Teri A Ansell, Danielle T Ryan, Phaik-Sim Lim, Sanjiv Vij, Rebecca Acs, Nigel Fealy, Elizabeth H Skinner

**Affiliations:** Department of Physiotherapy, Monash Health, 246 Clayton Road, Clayton, Victoria 3168 Australia; Allied Health Research Unit, Monash Health, 400 Warrigal Road, Cheltenham, Victoria 3192 Australia; Department of Physiotherapy, Faculty of Medicine, Nursing and Health Science, Monash University, McMahons Road, Frankston, Victoria 3199 Australia; Department of Intensive Care, Monash Health, 246 Clayton Road, Clayton, Victoria 3168 Australia; Department of Physiotherapy, Monash Health, 135 David Street, Dandenong, 3175 Victoria Australia; Department of Intensive Care, Monash Health, 135 David Street, Dandenong, 3175 Victoria Australia; Department of Intensive Care, Austin Health, Studley Road, Heidelberg, Victoria 3084 Australia; Department of Physiotherapy, Western Health, Gordon Street, Footscray, Victoria, 3011 Australia

## Abstract

**Introduction:**

Despite studies demonstrating benefit, patients with femoral vascular catheters placed for continuous renal replacement therapy are frequently restricted from mobilization. No researchers have reported filter pressures during mobilization, and it is unknown whether mobilization is safe or affects filter lifespan. Our objective in this study was to test the safety and feasibility of mobilization in this population.

**Methods:**

A total of 33 patients undergoing continuous renal replacement therapy via femoral, subclavian or internal jugular vascular access catheters at two general medical-surgical intensive care units in Australia were enrolled. Patients underwent one of three levels of mobilization intervention as appropriate: (1) passive bed exercises, (2) sitting on the bed edge or (3) standing and/or marching. Catheter dislodgement, haematoma and bleeding during and following interventions were evaluated. Filter pressure parameters and lifespan (hours), nursing workload and concern were also measured.

**Results:**

No episodes of filter occlusion or failure occurred during any of the interventions. No adverse events were detected. The intervention filters lasted longer than the nonintervention filters (regression coefficient = 13.8 (robust 95% confidence interval (CI) = 5.0 to 22.6), *P* = 0.003). In sensitivity analyses, we found that filter life was longer in patients who had more position changes (regression coefficient = 2.0 (robust 95% CI = 0.6 to 3.5), *P* = 0.007). The nursing workloads between the intervention shift and the following shift were similar.

**Conclusions:**

Mobilization during renal replacement therapy via a vascular catheter in patients who are critically ill is safe and may increase filter life. These findings have significant implications for the current mobility restrictions imposed on patients with femoral vascular catheters for renal replacement therapy.

**Trial registration:**

Australian and New Zealand Clinical Trials Registry ACTRN12611000733976 (registered 13 July 2011)

**Electronic supplementary material:**

The online version of this article (doi:10.1186/cc14001) contains supplementary material, which is available to authorized users.

## Introduction

Acute renal failure occurs in 5.5% to 6.0% of patients admitted to the intensive care unit (ICU), with almost three-fourths of these patients requiring the institution of continuous renal replacement therapy (CRRT) via temporary double-lumen vascular catheters [1]. Historically, patients with femoral vascular catheters have been restricted to bed rest [2, 3] to avoid catheter dislodgement, infection and thrombosis [4]. Patient movement may alter fluid dynamics, pressures and blood flow of the CRRT circuit [5]. In contrast, immobilization protocols may increase the risk of thrombosis and embolism [6]. Early mobilization in the ICU is generally safe [7] on the basis of an increasing evidence base [8–12]; in the context of evolved understanding of post-ICU syndrome [13–16], however, there are still specific clinical scenarios in which the safety and feasibility of mobilization has not been established. Moreover, CRRT is frequently present (in up to 9% of sessions) [3] in patients most likely to benefit (for example, those on mechanical ventilation for more than 48 hours).

The presence of femoral catheters is a considerable barrier to early mobilization [17]. Although mobilization in the presence of femoral arterial catheters is safe [18, 19], delivery of CRRT via femoral catheters precludes hip flexion in practice and research [3]. Researchers in several recent studies have reported data on the safety and feasibility of mobilization in patients with femoral catheters (including arterial, venous and haemodialysis) [5, 18–20], but none have reported CRRT data specifically during mobilization. Maintenance of the filter circuit is important, as premature disconnection results in loss of blood, increased nursing workload and increased costs [21]. Filter life is also an important indicator of CRRT efficacy [22]. The specific effects of mobilization on the vascular catheter, circuit pressures, fluid dynamics and blood flow in patients receiving CRRT via dual-lumen femoral vascular catheters are uncertain. Therefore, our objective in this study was to test the safety and feasibility of mobilization in ICU patients with femoral vascular catheter placement during CRRT.

## Material and methods

### Design, setting and participants

This prospective cohort study (Australian and New Zealand Clinical Trials Registry Number ACTRN12611000733976) was conducted between August 2011 and August 2012 in the 21-bed tertiary ICU at Monash Medical Centre and the 14-bed tertiary ICU at Dandenong Hospital, both of which are in Victoria, Australia. In the absence of empirical data on which to base the sample size, a convenience sample of 40 participants was selected. The institutional ethical review board responsible for both sites (Monash Health, Melbourne, Australia) approved the study at both sites. Informed consent was obtained from participants or their surrogate decision-makers. Participants were eligible if admitted to the ICU with the insertion of a vascular catheter for CRRT. Patients were excluded if they were receiving sustained low-efficiency dialysis or CRRT via permanent vascular access.

### Exclusion and cessation criteria

Passive group patients were ineligible to participate in the intervention if they met any of the following criteria:

-  Extreme agitation or confusion (Richmond Agitation–Sedation Scale +3 or +4 [23])
-  Heart rate >160 or <40 beats/min or new arrhythmia
-  Limb movement restricted for reasons other than the presence of the vascular catheter

Low-level or high-level group patients were ineligible for the reasons listed above or if they met any of the following criteria:

-  Mean arterial blood pressure <60 mmHg or >120 mmHg
-  >10 μg/min noradrenaline (or equivalent)
-  Fraction of inspired oxygen >0.6 and/or partial pressure of oxygen <65 mmHg
-  Peripheral oxygen saturation <85% or drop >10% from resting level
-  Respiratory rate >35 breaths/min
-  Temperature >38.5°C
-  Drowsy, unable to follow commands
-  New-onset chest pain with suspected cardiac cause

The intervention was ceased if these criteria were met without recovery in 2 minutes. Any CRRT alarms during the intervention were assessed and responded to by the bedside nurses. The intervention was then continued in consultation with the nurse after troubleshooting of the machine alarms was complete. If the CRRT alarms could not be resolved by the bedside nurse within 2 minutes and were thought to be associated with the intervention, mobilization was ceased.

### Procedure

Routine baseline data on the primary outcomes were recorded prior to study recruitment for 19 additional patients to monitor the Hawthorne effect. Participants were screened by treating ICU physiotherapists daily on weekdays. CRRT was generally delivered via continuous venovenous haemodiafiltration (CVVHDF) using Prismaflex ST100 filters (Gambro Lundia AB, Lund, Sweden) at a dialysate rate of 20 ml/kg/h, a replacement fluid rate of 15 ml/kg/h (delivered after the filter) and an effluent fluid removal rate of 50 to 100 ml/h with primarily Lactasol™ or Hemosol™ (Gambro Lundia AB).

### Intervention

The movement on vascular catheter evaluation (MOVE) intervention was delivered by senior treating ICU physiotherapist(s) at three different levels (passive, low-level physical function, high-level physical function), depending on the participant’s ability. No training of staff was required to deliver the intervention, as mobilization activities formed part of usual care in the study sites. A single intervention of 20 minutes (five positions for 4 minutes each) was delivered to reflect an effective clinical treatment dosage [9]. Prior to the intervention, investigators checked vascular catheter security and suturing. The following were the three intervention levels and details:

1. *Passive:* (a) Unable to participate (for example, sedation, low Glasgow Coma Scale score, severe weakness); (b) supine, sustained hip flexion (45°), supine, repeated-movement hip flexion (45°), supine.
2. *Low-level*: (a) Able to participate, assessed as likely unable to stand; (b) supine, repeated hip flexion (45°), supine, sitting on the edge of the bed, supine.
3. *High-level*: (a) Able to participate, assessed as likely being able to stand (with or without assistance); (b) supine, standing, marching on the spot, sitting on the edge of the bed, supine.

### Measurement

The following data were recorded on the day of intervention and daily thereafter for at least three further filters, until the intervention vascular catheter was removed or the patient was discharged from the ICU, whichever occurred first: age, sex, severity of illness (based on Acute Physiology and Chronic Health Evaluation (APACHE II and III) scores), mechanical ventilation, vascular catheter type, site, daily pathology (for example, platelets, international normalized ratio (INR), activated partial thromboplastin time (aPTT)), non-intervention-related position changes (daily) and sedation and delirium scores. Sedation and delirium were assessed using the Richmond Agitation–Sedation Scale [23] and the Confusion Assessment Method for the ICU [24, 25].

The primary outcome measure was the occurrence of adverse events during or after interventions, defined *a priori* as the following:

-  Vascular catheter dislodgement (assessed by visual inspection)
-  Filter circuit clotting or disruption (assessed by circuit disconnection)
-  Bleeding, haematoma at the vascular catheter site (assessed by visual inspection and medical and nursing documentation)
-  Clinical suspicion of thrombosis (vascular observations recorded every 2 hours postintervention, medical documentation, radiology for ultrasound referral)
-  Arrhythmia (assessed by visual inspection of electrocardiogram and medical documentation)

The following secondary outcome measures were used:

-  Filter life (measured from filter commencement to disconnection as documented by nursing staff (1:1 ratio))
-  Intervention feasibility (measured by filter alarm rates, pressures (access, return, transmembrane), blood flow recorded each minute from the digital output screen (Prismaflex))

Additional secondary measures included nursing workload and nurses’ concerns about filter disconnection (see Additional file 1 for more details on methods and results).

### Data analysis

The reason for cessation of filtration was recorded (either elective or not), and elective cessation filters were excluded from the filter life analyses. Descriptive statistics (median (IQR) or mean (SD)) were calculated as appropriate (Kolmogorov-Smirnov test). Linear regression analyses clustered by individual participant (to account for multiple filters within individual participants) and robust variance estimates were used to compare filter lifespan for filters where the participant was exposed to an intervention as opposed to filters where no intervention was provided. The number of filters that participants received was adjusted for by including the filter numbers (first filter = 1, second filter = 2 and so forth) in an interaction term with the intervention term in the analysis. Subgroup analyses separating data on the basis of vascular catheter site (femoral vs nonfemoral) and intervention group (passive vs low-level vs high-level) were performed. Sensitivity analyses were conducted to determine whether differences in aPTT, INR, platelets, non-intervention-related position changes (frequency), APACHE III scores and number of CRRT alarms during intervention influenced the primary analyses. In these sensitivity analyses, covariates were added to the regression models to examine their influence on the statistical significance of the MOVE intervention. Missing data were excluded listwise from analyses. Statistical analysis was performed using IBM SPSS Statistics™ 20 version 20.0.0 (SPSS, Chicago, IL, USA) and STATA/SE version 12 (StataCorp LP, Austin, TX, USA). *P* < 0.05 was accepted as the level of statistical significance.

## Results

**Figure 1:**
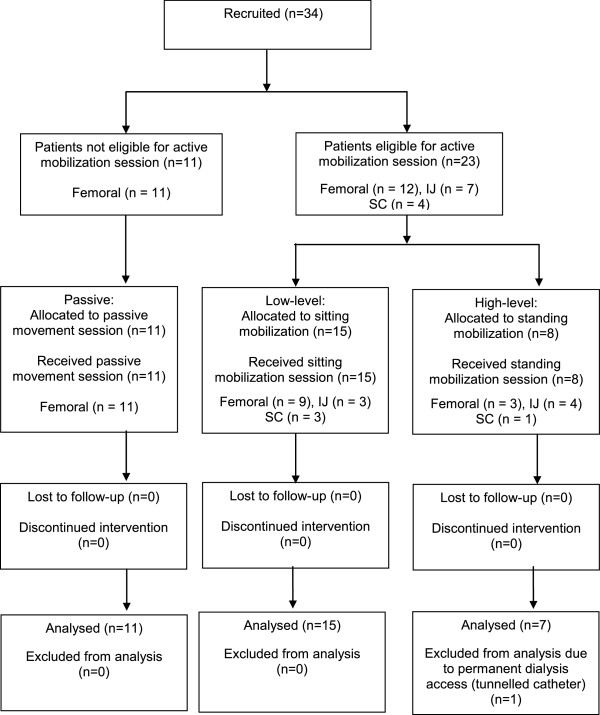
**Flowchart of participants through the study.** IJ: Internal jugular; SC: Subclavian.

In the analyses, 34 patients were included and 1 was excluded (Figure 1). Recruitment was ceased without six high-level intervention participants, as those meeting the relevant inclusion criteria were present in much lower than anticipated numbers. No patients died in the ICU. Twenty-three (17.2%) of one hundred thirty-four femoral filters and fourteen (23.0%) of sixty-one nonfemoral filters were excluded from filter life analyses (elective cessation). The sample was broadly representative of a general ICU cohort (Table 1). None of the filters were planned for disconnection on the intervention day. The vascular catheter was sutured upon site check prior to the intervention in all patients. Eleven, sixteen and six patients received a single passive, low-level or high-level intervention, respectively (Figure 1), with mean (SD) treatment duration of 19 (±3) minutes. The median (IQR) days of follow-up was 4 (2 to 6).

**Table 1:** **Demographic and clinical details of the sample at the time of the intervention** ^**a**^

Variable	Baseline group (***n*** = 19)	Intervention group (***n*** = 33)	Femoral (***n*** = 23)	Nonfemoral (***n*** = 10)
Age, yr	63.6 (13.6)	63.7 (14.8)	63.7 (14.1)	63.6 (17.2)
Sex, % male	79%	61%	65%	50%
BMI	28.0 (5.1)	29.3 (10.6)	31.7 (11.2)	23.9 (7.0)
Diagnosis, %				
Cardiogenic shock/cardiac	26%	33%	30%	40%
Septic shock/sepsis/MOF	47%	43%	44%	40%
Renal/metabolic/electrolyte	21%	15%	17%	10%
Haemorrhagic shock/haematoma	0%	9%	9%	10%
Vascular surgery	5%	0%	0%	0%
APACHE II score	–	26.1 (7.2)	25.7 (6.8)	26.9 (8.2)
APACHE III score	98.3 (26.9)	93.8 (24.9)	91.1 (23.1)	100.0 (29.1)
ICU length of stay, days	10.2 (6.9)	15.0 (10.0)	15.4 (9.3)	14.1 (11.8)
MV, %	74%	76%	74%	80%
MV hours, median (IQR)	78 (0 to 153)	76 (0 to 267)	89 (0 to 295)	63 (30 to 190)
Site^b^, %				
Femoral	63%	70%	100%	0%
Internal jugular	37%	21%	0%	70%
Subclavian	0%	9%	0%	30%
Catheter type^c^, *n* (%)				
Dolphin Protect	13 (68%)	19 (58%)	13 (57%)	6 (55%)
Niagara™	5 (26%)	9 (27%)	7 (30%)	2 (18%)
Arrow-Howes™	1 (5%)	5 (15%)	3 (13%)	1 (9%)
Intervention filter type, %				
Prismaflex ST100	N/A	94%	91%	100%
Intervention filter anticoagulation, %				
Heparin	N/A	27%	26%	30%
Citrate	N/A	6%	4%	10%
Saline	N/A	0%	0%	0%
Regional circuit	N/A	52	48%	60%
Other	N/A	3%	4%	0%
Nil	N/A	12%	17%	0%
FBE on intervention filter, median (IQR)				
Hb, g/dl	N/A	89 (85 to 94)	88 (81 to 91)	93 (90 to 103)
Platelets, 10^3^ U/L	N/A	125 (60 to 200)	121 (59 to 174)	179 (60 to 252)
INR	N/A	1.1 (1.1 to 1.4)	1.2 (1.1 to 1.4)	1.1 (1.1 to 1.3)
aPTT	N/A	39 (34 to 52)	40 (34 to 55)	34 (33 to 44)
RASS, median (IQR)	N/A	-1 (-4 to 0)	-2 (-4 to 0)	-1 (-1 to 0)
CAM-ICU positive, %	N/A	42%	39%	50%
Hospital LOS in days, median (IQR)	23 (9 to 31)	31 (21 to 57)	29 (21 to 57)	37 (15 to 57)
Hospital mortality, %	42%	15%	17%	10%

^a^aPTT, Activated thromboplastin time; BMI: Body mass index; CAM-ICU: Confusion Assessment Method for the ICU; FBE: Full blood examination; Hb: Haemoglobin; ICU: Intensive care unit; INR: International normalized ratio; IQR: Interquartile range; LOS: Length of stay; MOF: Multiorgan failure; MV: Mechanical ventilation; N/A: Not applicable; RASS: Richmond Agitation–Sedation Scale. ^b^Femoral (14 in right, 9 in left), intrajugular (6 in right, 1 in left) and subclavian (3 in left, 1 in right). ^c^Dolphin Protect (13-French gauge, 25 cm; Gambro, Hechingen, Germany), Niagara™ (13.5-French gauge; Bard Access Systems, Salt Lake City, UT, USA), Arrow-Howes™ triple-lumen (13-French, 20 cm; Teleflex, Research Triangle Park, NC, USA). Data are mean (SD) unless otherwise specified. Percentages may not add up to 100% due to rounding.

### Safety

No adverse events occurred during or following the interventions. One participant had a pulmonary artery catheter placed (in the low-level group), and no arrhythmias were associated with the interventions.

### Filter life

**Figure 2:**
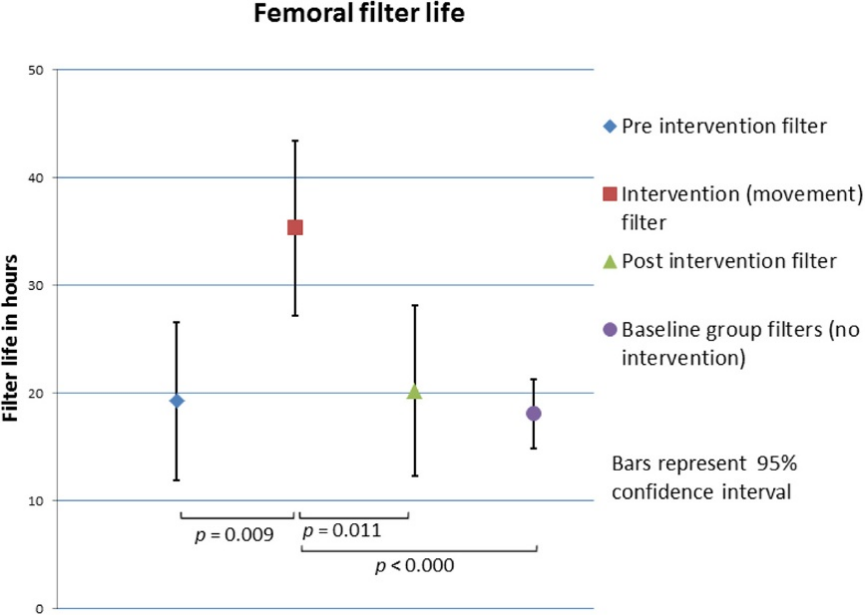
**Comparison of femoral filter life.** Preintervention filters are those that patients had prior to recruitment and intervention. Intervention filters are those in place during the time the intervention took place. Postintervention filters are those placed after the intervention filter.

Mean filter life at the time of the intervention was 19.5 hours (SD ±13.8), with no difference observed between femoral and nonfemoral filters (21.6 (15.1) hours vs 15.3 (9.9) hours, *P* = 0.45). Filters lasted for a mean of 17.4 (SD 12.7) hours after intervention. Intervention filters lasted longer than nonintervention filters (regression coefficient = 13.8, robust 95% confidence interval (CI) = 5.0 to 22.6, *P* = 0.003). A difference was also found in the femoral filter subgroup (regression coefficient = 15.7, robust 95% CI = 4.6 to 26.7, *P* = 0.008), but not in the nonfemoral access filter subgroup (regression coefficient = 9.2, robust 95% CI = -6.0 to 24.4, *P* = 0.20) (Figure 2).

An increasing effect of the MOVE intervention on filter life was evident in the higher the number of previous filters at the time of intervention (filter number × MOVE interaction effect: regression coefficient = 3.5, robust 95% CI = 0.3 to 6.6, *P* = 0.03). The effect of MOVE was approximately a 3-hour increase in filter life per filter already placed in the patient.

A higher number of daily position changes was associated with higher filter life in the overall cohort (regression coefficient = 2.0, robust 95% CI = 0.6 to 3.5, *P* = 0.007) and the femoral filter subgroup (regression coefficient = 2.0, robust 95% CI = 0.5 to 3.6, *P* = 0.01), but not in the nonfemoral filter subgroup (regression coefficient = 1.9, robust 95% CI = -1.7 to 5.4, *P* = 0.27) (Table 2). Alarm frequency during interventions was associated with a shorter filter life in the overall cohort (regression coefficient = -3.1, robust 95% CI = -5.0 to -1.2, *P* = 0.003) and the femoral filter subgroup (regression coefficient = -7.4, robust 95% CI = -13.0 to -1.8, *P* = 0.01), but, again, not in the nonfemoral filter subgroup (regression coefficient = -2.3, robust 95% CI = -4.6 to 0.0, *P* = 0.05). The passive intervention had a significant role in increasing filter life in the femoral filter subgroup, but only INR and aPTT were associated with filter life in the nonfemoral filter subgroup (Table 2). There were no significant differences in aPTT, INR, platelet count or rate of non-intervention-related position changes between the femoral filter intervention and nonintervention groups (Additional file 2). Addition of these covariates to the sensitivity analyses did not influence the effect of the MOVE intervention.

**Table 2:** **Subgroup and sensitivity analyses of filter life and possible confounders** ^**a**^

Group	Factors	Regression coefficient (robust 95% CI)	***P*** value
Baseline (*n* = 19)	INR	0.8 (-1.4 to 3.0)	0.44
	aPTT	0.1 (-0.3 to 0.5)	0.63
	Platelets	0.0 (0.0 to 0.1)	0.09
	Positional changes	1.2 (-0.4 to 2.9)	0.13
	APACHE III	-0.1 (-0.3 to 0.0)	0.09
Overall cohort (MOVE intervention) (*n* = 33)	MOVE intervention	13.8 (5.0 to 22.6)	0.003^b^
Overall subgroup and sensitivity analyses	Passive movements	20.0 (5.4 to 34.6)	0.01^b^
	SOEOB	5.8 (-10.7 to 22.3)	0.46
	MOS	18.3 (-1.3 to 37.9)	0.06
	INR	9.3 (-2.0 to 20.5)	0.10
	aPTT	0.0 (-0.2 to 0.2)	0.86
	Platelets	0.0 (-0.1 to 0.0)	0.10
	Positional changes	2.0 (0.6 to 3.5)	0.007^b^
	APACHE III	0.1 (0.0 to 0.3)	0.02^b^
	Alarms	-3.1 (-5.0 to -1.2)	0.003^b^
Femoral subgroup (*n* =23)	MOVE intervention	15.7 (4.6 to 26.9)	0.008^b^
Femoral subgroup and sensitivity analyses	Passive movements	20.0 (5.4 to 34.6)	0.01^b^
	SOEOB	8.5 (-20.6 to 37.7)	0.51
	MOS	20.5 (-19.9 to 60.9)	0.16
	INR	7.6 (-4.9 to 20.0)	0.22
	aPTT	-0.1 (-0.2 to 0.1)	0.38
	Platelets	0.0 (-0.1 to 0.0)	0.29
	Positional changes	2.0 (0.5 to 3.6)	0.01^b^
	APACHE III	0.6 (-0.1 to 0.2)	0.42
	Alarms	-7.4 (-13.0 to -1.8)	0.01^b^
Nonfemoral subgroup (*n* = 10)	MOVE intervention	9.2 (-6.0 to 24.4)	0.20
Nonfemoral subgroup and sensitivity analyses	Passive movements	N/A	–
	SOEOB	1.0 (-12.8 to 14.8)	0.86
	MOS	20.6 (-23.3 to 64.4)	0.23
	INR	26.4 (7.5 to 45.3)	0.01^b^
	aPTT	0.5 (0.1 to 0.9)	0.03^b^
	Platelets	-0.1 (-0.1 to 0.0)	0.08
	Positional changes	1.9 (-1.7 to 5.4)	0.27
	APACHE III	0.2 (0.0 to 0.5)	0.05
	Alarms	-2.3 (-4.6 to 0.0)	0.05

^**a**^APACHE III: Acute Physiology and Chronic Health Evaluation III; aPTT: Activated partial thromboplastin time; CI: Confidence interval; INR: International normalized ratio; MOS: Marching on the spot; MOVE: Movement on vascular catheter evaluation intervention; N/A: Not applicable; SOEOB: Sitting on edge of bed. Electively ceased filters were excluded from analysis. Twenty-three (17.2%) of one hundred thirty-four femoral filters and fourteen (23.0%) of sixty-one nonfemoral filters were excluded from the filter life analyses, as they were electively ceased. Units of measurement for filter life are hours. Position changes are measured as number per day. Alarms are the number of alarms during the intervention session. ^b^Statistically significant difference.

### Feasibility

No filter alarms sounded during interventions on 20 occasions (61% of the time). The machine alarmed a median of 0.0 (interquartile range = 0 to 2, range = 0 to 10) times during interventions. No differences in access, return or transmembrane (TM) pressures were observed in any group between the final and first phases (Additional file 3). There was a drop in access pressure during the sitting on edge of bed phase accompanied by a rise in TM pressure in the low-level group. There was an increase in access, return and TM pressures during the standing and marching phases in the high-level group, which returned to preintervention pressures during the final period (Additional file 3).

## Discussion

Mobilization of patients with femoral vascular catheters receiving CRRT in the ICU was safe and feasible. The intervention did not result in vascular catheter dislodgement, haematoma or bleeding, and there were no detectable clinical sequelae, including suspected thrombosis or filter circuit disruption. Average pressures did not approach circuit failure definitions (TM pressure >250 mmHg and access pressure >200 mmHg [26]) in any intervention group. These findings have significant implications for clinical practice situations where patients on CRRT are unnecessarily restrained from movement because of the perceived importance of these restrictions to maintaining filter patency and filter life and reducing mortality [27]. Interruptions in CRRT impact the dose of therapy delivered as well as clinical outcomes [27]. Testing mobilization during CRRT is critical, given that the opportunity to mobilize off CRRT can be minimal (minimum 16 hours required to maintain urea and creatinine, with a median time of 3 hours daily off filtration [22]), whereas time off CRRT can occur when it is impractical to mobilize (for example, overnight). Historically, contraindications to mobilization arose during an era of rigid medical plastics, which were associated with greater potential for vascular damage with movement. Although advances in materials [28] have resulted in more malleable catheters, manufacturers do not provide mobility specifications. These findings underscore the importance of empirically testing practices that have been accepted for many years. The presence of femoral vascular catheters for CRRT is a significant barrier to the delivery of early mobilization in the ICU [3, 17], as demonstrated in this study, where nurses’ concerns about circuit disconnection rose significantly when they were informed that mobilization was to occur. Few researchers have reported mobilization data regarding patients with femoral catheters [5, 18, 29, 30]. Although no catheter-related adverse events occurred during mobilization with femoral catheters in two of these previous studies [18, 30], only six patients in one cohort received an intervention with CRRT femoral catheters *in situ*[18] and the mobility intervention delivered to patients with femoral dialysis catheters was not specified in the other [30]. In neither study did the investigators report filter life or whether the intervention occurred during CRRT. Our present study is the first in which the safety and feasibility of mobilizing patients undergoing CRRT were prospectively evaluated.

It was important to test the safety of hip flexion, as key early mobilization trials have included passive range of motion [8] and cycle ergometry [9] as rehabilitation components. Patients undergoing CRRT should no longer be precluded from early mobilization on the basis that a vascular catheter or CRRT is *in situ*. It should be noted that the ability of patients undergoing CRRT to stand and march appears to be limited. In this study, we were able to recruit only three participants *with femoral catheters* who were able to perform these activities in 12 months in two ICUs. Talley and colleagues found that only 1.8% of their cohort were able to stand and/or ambulate with assistance [5], although the functional benefit of walking in the ICU compared to standing, marching on the spot or sitting on the edge of the bed is unclear. This is likely due to high hospital mortality, which is reported to range from 55% to 51% [5, 31], with 28-day mortality of 41% [32], though few studies have investigated functional and quality-of-life outcomes in this population [33].

Another key finding, which requires further empirical testing given the small sample size in this study, is that mobilization extended filter life, which has clinical, cost and potential survival implications. Increasing filter life could reduce nursing workload, costs, blood loss and infection risk [5]. Reducing flow stasis has been suggested to improve filter life [20], and, as exercise increases blood volume flow in healthy individuals [34, 35], the mechanism of mobilization is plausible. Although few researchers have investigated blood flow and immobilization in critical illness, it has been hypothesized that inactivity-related vascular injury, venous pooling and microvascular dysfunction increase thromboembolism risk [36, 37]. There are published data demonstrating that exercise increases blood volume flow in healthy individuals [34, 35]. It is therefore plausible that increasing blood flow could reduce thrombosis in critically ill patients who are undergoing continuous renal replacement therapy. The results of the sensitivity analyses in our present study support this hypothesis, as passive hip flexion and positional changes improved filter life in the femoral subgroup, but not in the nonfemoral subgroup. Because mean peak blood flow is usually higher in the subclavian and internal jugular veins than in the femoral veins [38, 39], we hypothesize that catheters sited in the femoral veins may be more susceptible to the effects of stasis with immobility. This hypothesis is supported by the results of our sensitivity analyses, which demonstrate that the MOVE intervention and position changes were not significantly associated with filter life (but that INR and aPTT were) in the nonfemoral subgroup. There are also potential trends in the data that may be more thoroughly explored with larger sample sizes; for example, large regression coefficients that were not statistically significant were seen for marching on the spot and INR in the overall cohort sensitivity analyses. It is possible that different results would be found in patients undergoing continuous venovenous haemofiltration rather than CVVHDF, although in Australia the majority of CRRT delivery is via CVVHDF [31]. Generalizability of the results to other ICUs may be influenced by variation in CRRT practices, although filtration practices in the two centres in this study were largely reflective of Australasian ICUs.

### Limitations

This study is limited by its single-health-service design, although it was conducted at two sites. The sample size was small (albeit one of the largest to date in this field reported in the literature). The results may have been influenced by sampling error; however, it should be noted that the characteristics of the study sample were largely consistent with characteristics of the routine baseline sample. Large multicentre studies are warranted to confirm our findings and further strengthen our conclusions, in particular those pertaining to filter life. Delivery of CRRT was not standardized, and the filter failure criteria were not specified *a priori*. The reason for filter cessation was not always recorded and could have been biased by nursing staff, although the average filter life of the nonintervention filters during the study period was the same as that of the baseline filters and less than half the nursing staff knew that their patients had mobilized during CRRT. Despite this, the main previously reported determinants of filter life were comparable between femoral and nonfemoral filters. A single intervention session was delivered to each patient, and it was not possible to examine possible dose–response relationships between duration of mobilization and filter life, because the intervention duration was standardized. In future studies, researchers could investigate a possible dose–response relationship between mobilization and filter life.

## Conclusions

Mobilization during CRRT via a vascular catheter in patients who are critically ill is safe and may increase filter life. Given the established benefit of early mobilization in the critical care population, early mobilization should be considered as part of the management of patients undergoing CRRT. Stasis secondary to immobility may contribute to the life of the haemodiafiltration circuit. Large multicentre studies are warranted to confirm the findings of our study and further strengthen our conclusions, particularly those pertaining to filter life.

## Key messages

-  Early mobilization improves health outcomes following admission to the ICU, including ventilation duration, length of stay and delirium. However, patients undergoing CRRT are frequently precluded from participation in early mobilization because of concerns about catheter safety and filter circuit patency. There are no previous studies in which researchers have reported data on filter circuit patency and filter life associated with early mobilization.
-  This study is the first in which filter life data were recorded for patients undergoing CRRT via a vascular catheter in the ICU. Mobilization was found to be safe and associated with no adverse events, and we found an increase in filter life in the group with femoral catheters.
-  Our presently reported work will have a significant impact on clinical medicine, as it provides empirical data suggesting that restrictions on mobilization imposed on patients undergoing CRRT are detrimental to filter life, which has a direct impact on the success of the therapy.
-  Our present research suggests that stasis of blood influences filter life, which may be a significant contributor to ICU morbidity and mortality in this population. This concept remains unexplored in the ICU literature.
-  Our findings have significant implications for the clinical management and morbidity of patients undergoing CRRT in critical care.

## Electronic supplementary material

Additional file 1: **Nursing workload and nursing concern about filter disconnection.** Includes methods and results of the effect of the intervention on nursing workload and nursing perception of likelihood of filter circuit discontinuation. (DOCX 23 KB)

Additional file 2: **Characteristics of filters by intervention group and access site.** Includes clinical data of baseline, intervention and nonintervention filters (femoral and nonfemoral). (DOCX 19 KB)

Additional file 3: **Mean CVVHDF filter parameters during intervention in patients with femoral catheters.** Includes variations in access and transmembrane pressure during the three levels of intervention: passive (hip flexion), low-level (hip flexion and sitting on edge of bed) and high-level (sitting on edge of bed, standing and marching on spot). (DOCX 996 KB)

## Abbreviations

aPTT: Activated partial thromboplastin time
CI: Confidence interval
CRRT: Continuous renal replacement therapy
CVVHDF: Continuous venovenous haemodiafiltration
ICU: Intensive care unit
IJ: Internal jugular
INR: International normalized ratio
IQR: Interquartile range
MOS: Marching on the spot
MOVE: Movement on vascular catheter evaluation
SC: Subclavian
SOEOB: Sitting on the edge of the bed
TM: Transmembrane.
