# Supplementary material for: Early mobilization on continuous renal replacement therapy is safe and may improve filter life
Source: Crit Care. 2014 Jul 28;18(4):R161. doi: 10.1186/cc14001 (PMC4262200; doi:10.1186/cc14001)
Supplement: Supplementary file 1 — Additional file 1: Nursing workload and nursing concern about filter disconnection. Includes methods and results of the effect of the intervention on nursing workload and nursing perception of likelihood of filter circuit discontinuation. (DOCX 23 KB) [file 13054_2014_2717_MOESM1_ESM.docx]

**Additional File 1.**

**Nursing workload and nursing concern about filter disconnection.**

*Methods*

Nursing workload was measured using the NASA-TLX and the Nine Equivalents of Nursing Manpower use score (NEMS) during the intervention shift and on the subsequent shift. Originally developed to measure workload in aviation [[1](#_ENREF_1)], the NASA-TLX is reliable, valid and easy to administer measurement of subjective workload perception in ICU nurses [[2](#_ENREF_2)]. The NEMS measures the objective nursing workload and is easy to administer, reliable and valid [[3](#_ENREF_3), [4](#_ENREF_4)].

Nursing concern about filter disconnection was measured using a Likert scale of 1 = extremely concerned, 2 = very concerned, 3 = moderately concerned, 4 = mildly concerned and 5 = not at all concerned. Nurses rated their concern about filter disconnection prior to being informed of the mobilization plan and again once informed of the specific details of the intervention by the treating therapist.

*Analysis*

Nursing workload and concern were analyzed using paired t-tests and Fisher’s Exact Test. For analyses, the Likert categories of nursing concern were collapsed to concerned (rating = 1, 2, 3 or 4) vs. Unconcerned (rating = 5). Statistical analysis was performed using IBM SPSS Statistics™ 20 Version 20.0.0 (SPSS Inc., Chicago, IL, USA) and *p* < 0.05 was accepted as statistical significance.

*Results*

No differences in nursing workload were seen between the intervention and following shifts, as measured by either the NASA-TLX (mean (SD) 13.1 (3.1) vs. 12.2 (4.9), mean difference (95% CI) -0.93 (-3.6, 1.7), *p* = 0.46) or the NEMS (mean (SD) 33.4 (10.7) vs. 33.9 (10.6), mean difference (95% CI) 0.45 (-6.9, 6.0). A NASA-TLX score of 32.8 is equivalent to administering injections [[5](#_ENREF_5)] and the mean scores for both shifts were considerably lower, although the mean NEMS was higher than usual (mean 26.2, SD 9.4) [[3](#_ENREF_3)]. Nursing concern about the likelihood of the filter circuit clotting was heightened by informing nurses that the intervention was going to occur (61% vs. 76%, *p* < 0.001). The day following intervention, only 48% of nurses knew their patient had been mobilized on the filter the preceding day.

**References**

1. Hart SG, Staveland LE: **Development of NASA-TLX (Task Load Index): Results of empirical and theoretical research.** In: *Human Mental Workload*. Edited by Hancock PA, Meshkati N. Amsterdam: North Holland Press; 1988.

2. Hoonakker P, Carayon P, Gurses A, Brown R, McGuire K, Khunlertkit A, Walker JM: **Measuring Workload of Icu Nurses with a Questionnaire Survey: The Nasa Task Load Index (Tlx).** *IIE Trans Healthc Syst Eng* 2011, 1(2):131-143.

3. Reis Miranda D, Moreno R, Iapichino G: **Nine equivalents of nursing manpower use score (NEMS).** *Intensive Care Med* 1997, 23(7):760-765.

4. Rothen HU, Kung V, Ryser DH, Zurcher R, Regli B: **Validation of "nine equivalents of nursing manpower use score" on an independent data sample.** *Intensive Care Med* 1999, 25(6):606-611.

5. Burford E: **The analysis of the strain level and the predicted human error probability for critical hospital tasks.** Grahamstown: Rhodes University; 2012.
