# Supplementary material for: Early mobilization on continuous renal replacement therapy is safe and may improve filter life
Source: Crit Care. 2014 Jul 28;18(4):R161. doi: 10.1186/cc14001 (PMC4262200; doi:10.1186/cc14001)
Supplement: Supplementary file 2 — Additional file 2: Characteristics of filters by intervention group and access site. Includes clinical data of baseline, intervention and nonintervention filters (femoral and nonfemoral). (DOCX 19 KB) [file 13054_2014_2717_MOESM2_ESM.docx]

**Additional File 2.**

**Characteristics of filters by intervention group and access site (median (IQR) unless otherwise indicated).**

|  | **Baseline filters**  **n=69** | Baseline femoral filters  n=46 | Baseline non-femoral filters n=23 | **All intervention filters**  **n=33** | Femoral intervention filters  n=23 | Non-femoral intervention filters  n=10 | **All non-intervention filters**  **n=93** | Femoral non-intervention filters  n=65 | Non-femoral non-intervention filters  n=28 |
| --- | --- | --- | --- | --- | --- | --- | --- | --- | --- |
| Filter life^a^ (hours) | 22.3 (15.2) | 18.0 (11.1) | 32.6 (18.8) | 35.2 (17.2) | 35.3 (17.6) | 34.9 (17.4) | 21.4 (18.3) | 19.5 (16.3) | 25.8 (22.1) |
| Hb (g/dL) | 94 (87-103) | 95 (88-104) | 92 (84-99) | 89 (85-94) | 88 (81-91) | 93 (90-103) | 91 (85-96) | 89 (85-95) | 94 (82-99) |
| INR | 1.2 (1.0-1.6) | 1.2 (1.0-1.7) | 1.2 (1.1-1.6) | 1.1 (1.1-1.4) | 1.2 (1.1-1.4) | 1.1 (1.1-1.3) | 1.2 (1.1-1.4) | 1.2 (1.1-1.4) | 1.1 (1.0-1.2) |
| Platelets, x10^3^/UL | 145 (88-216) | 133 (85-212) | 149 (116-226) | 125 (60-200) | 121 (59-174) | 179 (60-252) | 111 (60-171) | 108 (59-149) | 137 (73-288) |
| APTT (seconds) | 36 (31-50) | 34 (30-48) | 38 (34-54) | 39 (34-52) | 40 (34-55) | 34 (33 – 44) | 37 (30 – 53) | 38 (30 – 52) | 37 (30 – 53) |
| Position changes/Day | 6 (3-7) | 6 (3-7) | 6 (4-7) | 6 (5-7) | 5 (4-7) | 8 (6-9)* | 5 (4-7) | 5 (3-7) | 6 (4-7)* |
| Anticoagulation method^b^ |  |  |  |  |  |  |  |  |  |
| Heparin | 13% | 15% | 9% | 27% | 26% | 30% | 24% | 24% | 25% |
| Citrate | 14% | 17% | 9% | 6% | 4% | 10% | 3% | 5% | 0% |
| Regional heparinisation | 32% | 37% | 22% | 52% | 48% | 60% | 38% | 35% | 46% |
| Other | 3% | 0% | 9% | 3% | 4% | 0% | 10% | 5% | 21% |
| Nil | 25% | 24% | 26% | 12% | 17% | 0% | 26% | 35% | 7% |

^a^Mean (SD), ^b^percentage. Percentages may not add up to 100% due to rounding. Hb, haemoglobin. Heparin, unfractioned heparin. INR, International Normalized Ratio. APTT, Activated Partial Thromboplastin Time. Regional Heparinization, unfractioned heparin infusion combined with protamine infusion. *Significant difference between groups, regression co-efficient [95% CI] 2 [1, 3], p*=*0.002.
