# Supplementary material for: Early mobilization on continuous renal replacement therapy is safe and may improve filter life
Source: Crit Care. 2014 Jul 28;18(4):R161. doi: 10.1186/cc14001 (PMC4262200; doi:10.1186/cc14001)
Supplement: Supplementary file 3 — Additional file 3: Mean CVVHDF filter parameters during intervention in patients with femoral catheters. Includes variations in access and transmembrane pressure during the three levels of intervention: passive (hip flexion), low-level (hip flexion and sitting on edge of bed) and high-level (sitting on edge of bed, standing and marching on spot). (DOCX 996 KB) [file 13054_2014_2717_MOESM3_ESM.docx]

**Additional File 3.**

**Mean CVVHDF filter parameters during intervention in patients with femoral catheters.**

Data presented as variations to the pre-intervention filter pressure measured at the beginning of the intervention session. Pressure parameters are compared within each filter to the pre-intervention parameters at time 0. The shaded region represent period of movement or mobilization, non-shaded region represent periods of rest or recovery. Error bars represent one standard deviation on either side of the mean. Each bar (95% confidence interval) represents average pressures of all participants calculated each minute.

*Access pressure during passive intervention*

*Access pressure during low-level intervention*

*Access pressure during high-level intervention*

*Transmembrane pressure during passive intervention*

*Transmembrane pressure during low-level intervention*

*Transmembrane pressure during high-level intervention*

Lost to follow-up (give reasons) (n= )

Discontinued intervention (give reasons) (n= )

Lost to follow-up (give reasons) (n= )

Discontinued intervention (give reasons) (n= )

Lost to follow-up (give reasons) (n= )

Discontinued intervention (give reasons) (n= )
